# Supplementary material for: Nasal respiration is necessary for ketamine-dependent high frequency network oscillations and behavioral hyperactivity in rats
Source: Sci Rep. 2020 Nov 4;10:18981. doi: 10.1038/s41598-020-75641-1 (PMC7642442; doi:10.1038/s41598-020-75641-1)
Supplement: Supplementary file 1 — Supplementary Figures. [file 41598_2020_75641_MOESM1_ESM.docx]

**Nasal respiration is necessary for ketamine-dependent high frequency network oscillations and behavioral hyperactivity in rats**

Jacek Wróbel, MSc^1^, Władysław Średniawa, MSc^1,2^, Gabriela Jurkiewicz, MSc^3^, Jaroslaw Zygierewicz, PhD^3^, Daniel K Wójcik, PhD^1,4^, Miles Adrian Whittington, PhD^5^, Mark Jeremy Hunt, PhD^1^

1. Nencki Institute of Experimental Biology, 3 Pasteur Street, 02-093 Warsaw, Poland

2. Faculty of Biology, University of Warsaw, 02-096, Warsaw, Poland

3. Faculty of Physics, University of Warsaw, 02-096 Warsaw, Poland

4. Faculty of Management and Social Communication, Jagiellonian University, 30-348 Cracow, Poland

5. University of York, Heslington, York, YO10 5DD, United Kingdom

Corresponding author: Mark Jeremy Hunt, Nencki Institute of Experimental Biology, 3 Pasteur Street, 02-093 Warsaw, Poland

Tel. (+48) 22 5892 138 Fax. (+48) 22 8225 342

Email: [m.hunt@nencki.edu.pl](mailto:m.hunt@nencki.edu.pl)

**
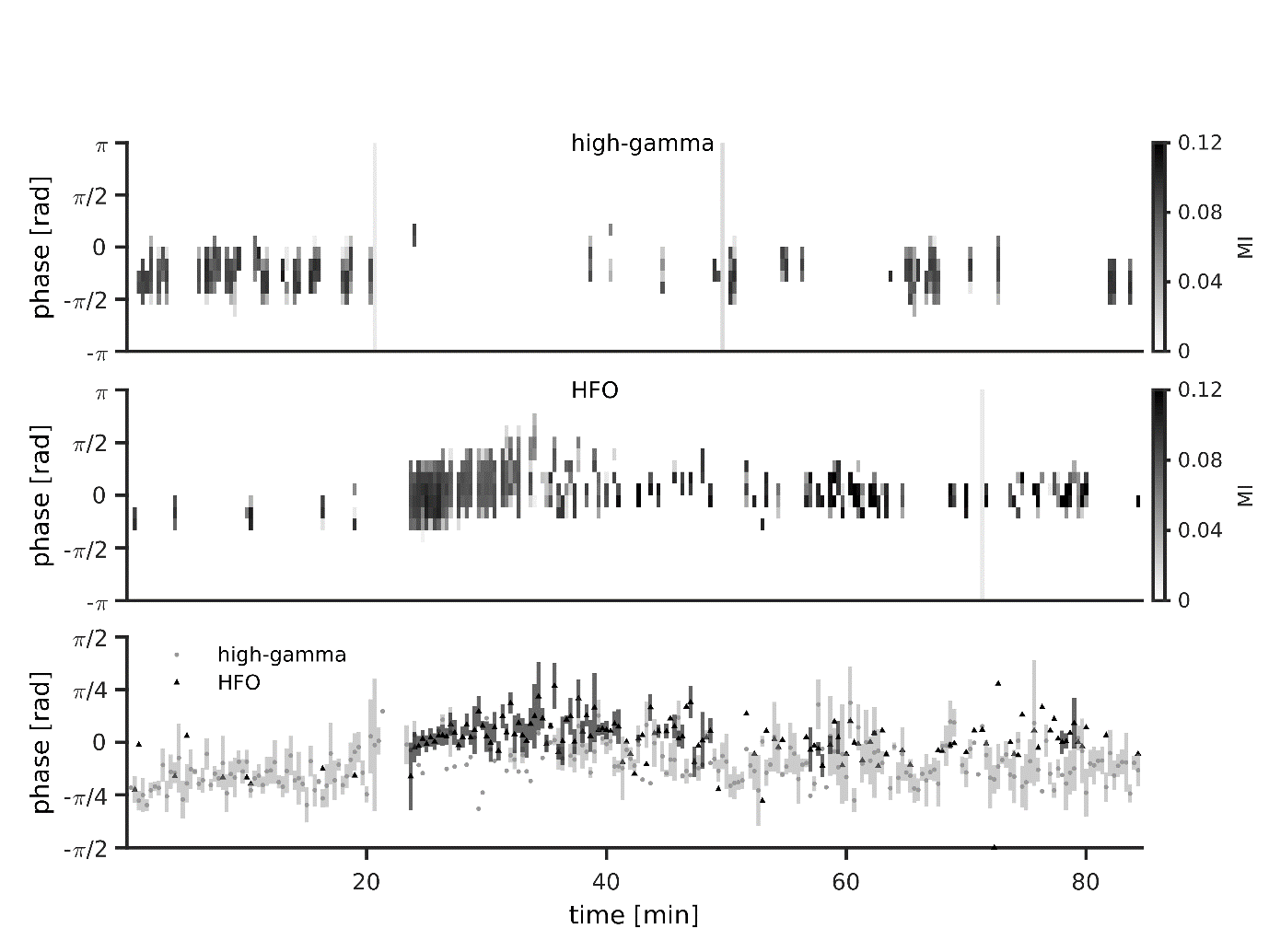
**

**Supplementary Figure S1. High-gamma and HFO lock to different phases of theta oscillations.** Example of the time course showing the effect of ketamine on the phase and strength of coupling of OB high-gamma (70-100 Hz, top) and HFO (130-180 Hz, middle) with respect to local theta. The bottom panel shows the mean and SEM for all rats. Injection of 20 mg/kg ketamine occurred around 20 min. Time courses show significant coupling only.


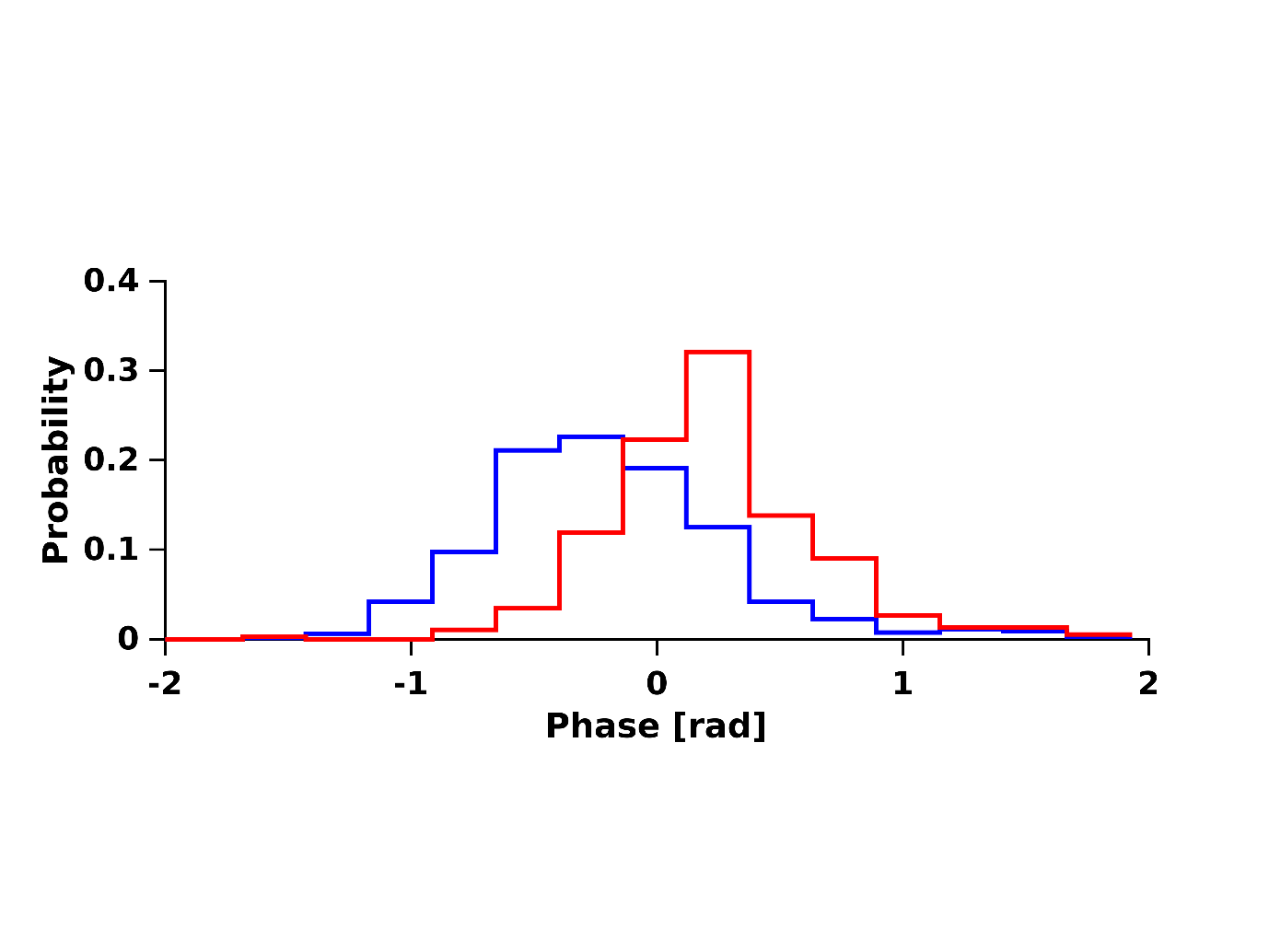


**Supplementary Figure S2. High-gamma and HFO occur at different phases of slow oscillations.** Histogram showing the distributions of coupled phase for HFO (red) and gamma (blue). The count number is normalized by a number of all samples resulting in probability of observation on vertical axis. The phase information is combined for all rats and all time points. Two-sample Kolmogorov–Smirnov test showing the distributions are statistically different p=7.0932e-45 with the Kolmogorov–Smirnov statistic = 0.4442.


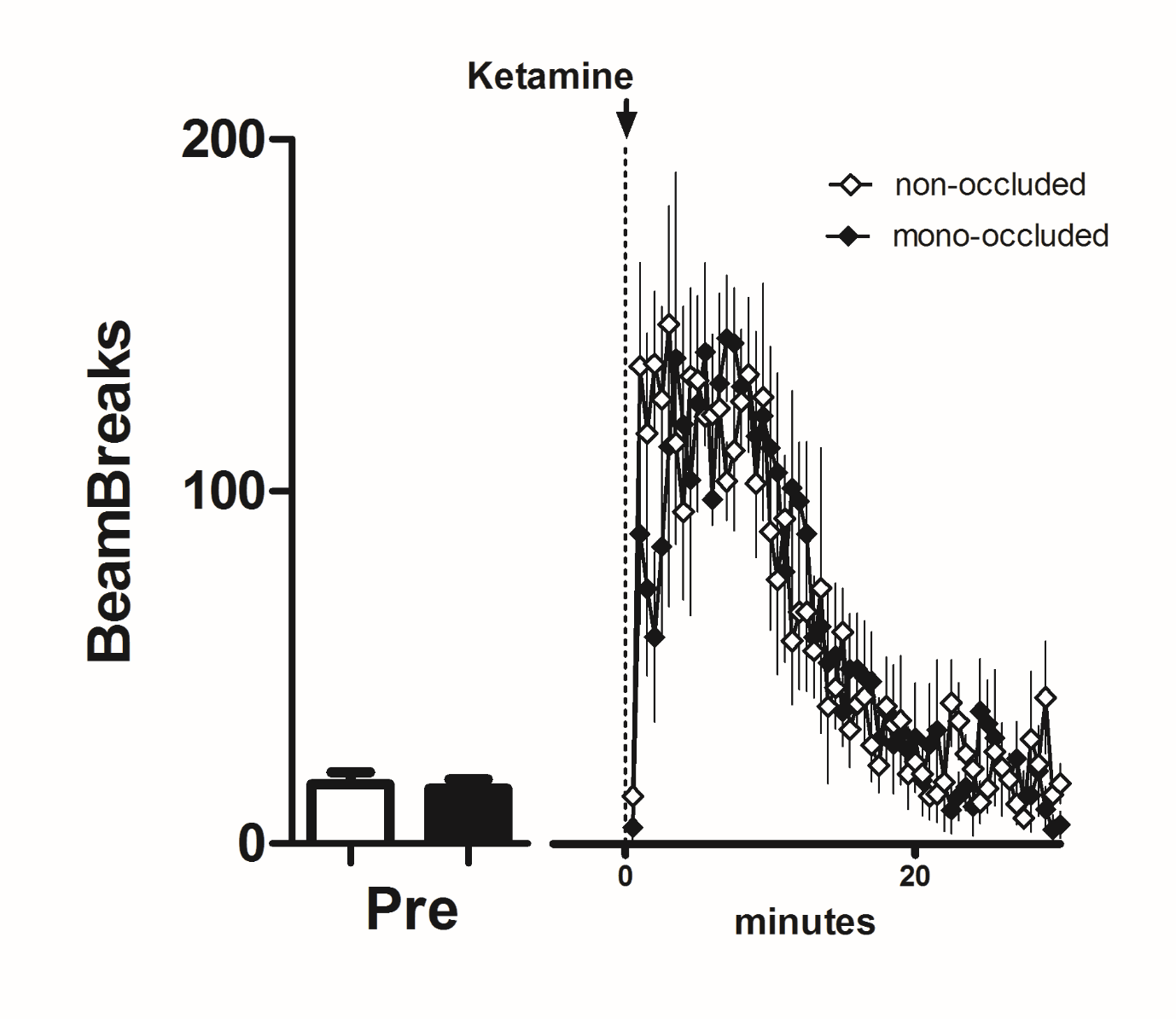


**Supplementary Figure S3.** **Unilateral naris occlusion does not affect ketamine-induced locomotion.** Time course of beam breaks after 20 mg/kg ketamine for mono-occluded naris rats and control non-occluded rats. Pre= pre-injection of ketamine.
